# Metabolic Parameters as Predictors for Progression Free and Overall Survival of Patients with Metastatic Colorectal Cancer

**DOI:** 10.1007/s12253-020-00865-5

**Published:** 2020-07-13

**Authors:** Zsuzsanna Nemeth, Wouter Wijker, Zsolt Lengyel, Erika Hitre, Katalin Borbely

**Affiliations:** 1 Chemotherapy B and Clinical Pharmacological Department of National Institute of Oncology, Ráth György utca 7-9, Budapest, 1122 Hungary; 2 Oncology Department of Szent Margit Hospital, Bécsi út 132, Budapest, H-1032 Hungary; 3 Auxiliis Pharma Ltd, Bokor utca 17, Budapest, H-1037 Hungary; 4 Pozitron Diagnostic Ltd, Hunyadi ut 9-11, Budapest, 1117 Hungary; 5 PET Ambulatory Department of National Institute of Oncology, Rath Gyorgy utca 7-9, Budapest, 1122 Hungary

**Keywords:** Standardized added metabolic activity, FDG-PET/CT, Interim, Metastatic colorectal cancer, Overall survival, Progression free survival, Response

## Abstract

We tested the prognostic relevance of metabolic parameters and their relative changes in patients with metastatic colorectal cancer (mCRC) treated with monoclonal antibody and chemotherapy. SUV_max_ (standardized uptake volume), SAM (standardized added metabolic activity) and TLG (total lesion glycolysis) are assessed with ^18^F-fluorodeoxyglucosepositron emission tomography and computed tomography (FDG-PET/CT) to evaluate total metabolic activity of malignant processes. Our purpose was to investigate the change of glucose metabolism in relation to PFS (progression free survival) and OS (overall survival). Fifty-three patients with mCRC with at least one measurable liver metastasis were included in this prospective, multi-center, early exploratory study. All patients were treated with first-line chemotherapy and targeted therapy. Metabolic parameters, like SUV_max_, SAM, normalized SAM (NSAM) and TLG were assessed by FDG-PET/CT, carried out at baseline (scan-1) and after two therapeutic cycle (scan-2). Our results suggested neither SUVmax nor TLG have such prognostic value as NSAM in liver metastases of colorectal cancer. The parameters after the two cycles of chemotherapy proved to be better predictors of the clinical outcome. NSAM after two cycles of treatment has a statistically significant predictive value on OS, while SAM was predictive to the PFS. The follow up normalized SAM after 2 cycles of first line oncotherapy was demonstrated to be useful as prognostic biomarkers for OS in metastatic colorectal cancer. We should introduce this measurement in metastatic colorectal cancer if there is at least one metastasis in the liver.

**Electronic supplementary material:**

The online version of this article (10.1007/s12253-020-00865-5) contains supplementary material, which is available to authorized users.

## Introduction

Colorectal cancer (CRC) is the third leading cause of cancer-related death. Colon cancer accounts for two thirds of all CRC cases and differs from rectal cancer in gender distribution and sites of metastases. About 60% of all patients with CRC develop metastatic disease, and the liver is by far the most common site of the spread [1]. Unfortunately, only a minority (approximately 25%) of these metastases are amenable to resection, while most of them are not resectable at the time of diagnosis [2]. An international panel of multidisciplinary experts [3] recommended to use high-quality magnetic resonance imaging (MRI) and/or computed tomography (CT) for mapping liver metastases preoperatively in patients with CRC. PET/CT was suggested for patients with a high risk for extrahepatic metastases, as this method is expected to have higher sensitivity compared to CT. It is already obvious, that the metabolic changes induced by chemotherapy in tumor cells are predictive of patient outcome and that PET/CT is more suitable for monitoring the response to therapy [4, 5] than other imaging modalities in targeted therapy in cancer. Current treatment standards of mCRC are based on chemotherapy combined with monoclonal antibody Typically, therapeutic response is assessed by CT after three to four cycles of therapy, but an earlier (after the first round of treatment, or even at the baseline, if possible) prediction of clinical outcome would be desirable, to guide further treatment regimens.

However, the prognostic value of baseline and early follow-up PET/CT parameters in rectal cancer and CRC remains debated. Some studies [6] showed that PET/CT changes after 2 months predicted overall survival (OS) and progression-free survival (PFS), others [4, 7] showed that PET/CT failed to reflect long-term outcome (OS), or questioned its sensitivity in the daily routine. A study of 44 patients with metastatic CRC (mCRC) did not find any correlation between maximum of the standardized uptake volume (SUV_max_) and KRAS exon 2 mutation [8], but others reported that PET/CT was useful for predicting KRAS/BRAF mutational status, respectively [9].

Considering the lack of consensus on the use of the metabolic data based on PET/CT examinations, the aim of this prospective study was to compare the available metabolic parameters and to establish the prognostic value of baseline and follow-up PET/CT for the long-term outcome in patients with mCRC.

## Materials and Methods

### Patients

Patients diagnosed with mCRC between April 2014 and November 2016 were enrolled in the current study. All patients were treated at Saint Margit Hospital (Budapest, Hungary) or at the National Institute of Oncology (Budapest, Hungary).

Inclusion criteria were the following: (1) patients with mCRC scheduled for first-line chemotherapy combined with monoclonal antibody therapy (bevacizumab or cetuximab) based on multidisciplinary board decision; (2) every patient was required to have at least one metabolically measurable metastatic lesion in the liver (> = 2 cm); (3) patients’ performance status should be less than ECOG two; (4) life expectancy of >8 weeks; and (5) signed informed consent.

Patients with (1) a history of allergic reactions to intravenous iodinated contrast agents, or (2) suffering from claustrophobia or (3) uncontrolled diabetes were excluded from our study. Those patients were also excluded who previously received chemo- or targeted therapy for their metastatic disease.

The study was approved by National Institute of Pharmacy and Nutrition (OGYÉI) and the Ethics Committee of the Medical Research Council (ETT-TUKEB) and complied with the Helsinki Declaration.

#### Treatment

Chemotherapy plus monoclonal antibody was administered according to current Hungarian guidelines.

Genetic testing of patients for somatic mutation in KRAS and NRAS is routinely applied in our patients [10]. Tumor tissue samples were investigated in some cases from primary site (if the metastases appeared at the same time with primary tumor) or from metastatic sites (if these metastases were metachron).

Methods used for testing the KRAS / NRAS mutations Genomic DNA from formalin-fixed, paraffin-embedded tissue (FFPET) – after deparaffinization – was extracted with the cobas® DNA Sample Preparation Kit, ROCHE.

Kras exon 2 (codon 12 and 13) and exon 3 (codon 61) mutation analysis was performed usingcobas® KRAS Mutation Test (Roche), on the cobas z 480 analyzer (Roche). Sensitivity of the method was 5%, specificity: 99%.

For screening the Nras exon 2 (codon 12 and 13) and exon 3 (codon 59 and 61) mutations, we used an in-house assay based on melting curve analysis on the LightCycler 2.0 instrument (Roche). Primers and FRET probes were purchased from IDT. Analytical sensitivity was 10%, specificity: 100%.

For screening the Kras exon 4 (codon 117 and 146) and NRAS exon 4 (codon 117 and 146) mutations, we used a HRM detection-based in-house assay, where primers and probes were from IDT, LC green was from BioFire Defense LTD, and the assay was carried out on the cobas z 480 analyzer (Roche). Analytical sensitivity was 10%, specificity: 100%.

Patients were categorized according to *KRAS* or *NRAS* mutation status into 2 groups: mutant *RAS* and wild-type *RAS*. Monoclonal antibody therapy was selected accordingly.

Monoclonal antibodies are dosed as follows: bevacizumab 5 mg/kg intravenously .or cetuximab 500 mg/m2, intravenously. Bevacizumab and cetuximab was applied in 35 patients and 18 patients, respectively. In addition to the monoclonal antibody therapy, patients were treated with chemotherapy: either FOLFOX4 (oxaliplatin 100 mg/m^2^/2h on day1, leucovorin 200 mg/m^2^/2h on day 1–2, 400 mg/m^2^/10min 5-FU on day1–2 and 5-FU continuous infusion 1200 mg/m^2^/46h) or FOLFIRI (irinotecan 180 mg/m^2^/90min on day1, leucovorin 200 mg/m^2^/2h on day 1–2, 400 mg/m^2^/10min 5-FU on day 1–2. and 5-FU continuous infusion 1200 mg/m^2^/46h) regimens were administered.

Treatment cycles were repeated every 14 days. All patients were treated with the same regimen as the first applied, until disease progression or if excessive toxicity was noted. After disease progression different second and third line treatment regimens were applied, according to the physician’s choice.

#### FDG-PET/CT Imaging

PET/CT scans were carried out at baseline (scan-1) and on day 21 (scan-2), after two cycles of combined chemotherapy. Patients were examined with PET/CT (Siemens Biograph TruePoint 6 HD, Siemens, Knoxville, US), according to routine oncological protocols. Patients fasted for at least 6 h (except diabetic patients, who fasted for 4 h) before examination. Uptake time was 60 ± 5 min in case of both scan-1 and scan-2. Low-dose, whole body CT scan (120 keV, 60 mA) preceded the PET imaging, which was started 7–10 min after the intravenous administration of 3.7 MBq FDG per kilogram body weight. PET raw data were iteratively reconstructed with proper correction for decay, dead-time, scatter, randoms and tissue attenuation with the help of the CT to display standardized (to body weight and injected activity) uptake values (SUV). PET/CT data were analyzed by two independent nuclear medicine specialists.

#### Image Analysis

PET/CT parameters which were measured in case of the metastatic liver lesions included maximum standardized uptake values (SUV_max_), total lesion glycolysis (TLG), standardized added metabolic activity (SAM), and normalized standardized added metabolic activity (NSAM). The SUV_max_ normalized to body weight was measured by the PMOD software (v3.310, Zürich, Switzerland). SAM seeks to determine the total metabolic activity above background due to tumor uptake while avoiding partial volume effect. It was calculated by the same formula as used by Mertens et al. [11]. Briefly, a first volume of interest (VOI, ie.VOI1) was drawn around the metastatic lesion in the liver. A second VOI (VOI2) was delineated around VOI1, directed to a small zone of homogeneous background. SAM was calculated as follows:

$$ \mathrm{SAM}=\mathrm{total}\ \mathrm{SUV}\ \mathrm{VOI}1-\left(\mathrm{mean}\ \mathrm{BG}\times \mathrm{volume}\ \mathrm{VOI}1\right), $$

where mean BG represents mean background activity, which was derived using the following formula:

$$ \mathrm{mean}\ \mathrm{BG}=\frac{\mathrm{total}\ \mathrm{SUV}\ \mathrm{VOI}2-\mathrm{total}\ \mathrm{SUV}\ \mathrm{VOI}1\ }{\mathrm{volume}\ \mathrm{VOI}2-\mathrm{volume}\ \mathrm{VOI}1} $$

in which total SUV is the product of the mean SUV and the respective volume. In patients with multiple liver metastases, SAM was calculated as the sum of the individual SAMs of the lesions.

Table 1 shows all metabolic parameters and their calculation methods for which the relation with OS and PFS was investigated.

**Table 1:** Metabolic variables investigated (1- scan1, 2- scan2)

Abbreviation	Explanation	Derivation
SUV _max_	The single liver lesion with the highest SUV_max_ value	
∑SAM	Summation of all existing lesions SAM values	∑SAM = SAM1 + SAM2 + SAMn…
NSAM	∑SAM normalized to the background	$\mathrm{NSAM}=\frac{\sum \mathrm{SAM}\ }{\mathrm{background}\ }$
TLG	Product of MTV (metabolic tumor volume) and SUV _mean_	TLG = SUV _mean_ × MTV
∆SUV_max_	The difference between SUV_max_ values	SUV_max_2 – SUV_max_1
∆SAM	The difference between ∑SAM values	∑SAM2 - ∑SAM1
∆NSAM	The difference between ∑NSAM values	∑NSAM2 -∑NSAM1
∆TLG	The difference between TLG values	TLG2 - TLG1
∆SUV%	The percentage change of the SUV _max_ value	$\frac{\mathrm{SUVmax}2-\mathrm{SUVmax}1}{\mathrm{SUVmax}1}\mathrm{x}100$
∆SAM%	The percentage change of the SAM value	$\frac{\mathrm{SAM}2-\mathrm{SAM}1}{\mathrm{SAM}1}\mathrm{x}100$
∆NSAM%	The percentage change of the NSAM value	$\frac{\mathrm{N}\ \mathrm{SAM}2-\mathrm{N}\ \mathrm{SAM}1}{\mathrm{N}\ \mathrm{SAM}1}\mathrm{x}100$
∆TLG%	The percentage change of the TLG values	$\frac{\mathbf{TLG2}-\mathbf{TLG}\mathbf{1}}{\mathbf{TLG1}}\ \mathbf{x}\mathbf{1}\mathbf{00}$

#### Response Assessment

Metabolic response was categorized according to the adapted EORTC (European Organization for Research and Treatment of Cancer) PET criteria [12]. The highest pre- and posttreatment SUV_max_, the percentage change of SUV_max_ (Table 1), percentage change of SAM, NSAM and TLG were also calculated. Patients with a reduction in SUV_max_ more than 25% were classified as responders, meanwhile, when an increase above 25% was found, patients were categorized as non-responders. Different thresholds were applied (30%, respectively) for SAM and TLG to classify the response rates, according to Mertens et al. [11]. As can be seen in Table 2, stable metabolic disease was also defined.

**Table 2:** Response criteria used in the evaluation after two cycles of systemic therapy

Response Category	EORTC criteria	Adapted for SAM, norm SAM and TLG
Responders		
Complete metabolic response	Disappearance of all lesions	Disappearance of all lesions
Partial metabolic response	Decrease>25% of SUV_max_	Decrease>30% of NSAM, norm SAM or TLG
Non-responders		
Stable metabolic disease	Increase <25% of SUV_max_ Decrease <15% of SUV_max_ No increase of the extension of FDG-uptake	Increase <30% Decrease <30% Of NSAM, NSAM or TLG
Progressive metabolic disease	New lesions Increase>25% of SUV_max_	New lesions Increase>30% of NSAM, NSAM or TLG

*EORTC* European Organisation for Research and Treatment of Cancer, *SAM* standardized added metabolic activity, *NSAM* normalized SAM, *TLG* total lesion glycolysis

#### Statistical Analysis

The analysis of the study data followed the principle of intention-to-treat. All applied statistical tests were two-sided and *p*-values<0.05 were considered significant.

OS and PFS was calculated from the first therapeutic cycle until date of death and CT confirmed progression, respectively. Right censoring was applied as per the last date of follow-up for the patients alive or for patients who did not show progression at the last follow up or at the end of the study. Log-rank analyses were used to assess the relationship between the clinical characteristics and PFS and OS. Cox regression was carried out with stepwise selection of variables. Variables with a *p* value smaller than <0.25 were selected in the model. 95% Wald and Likelihood confidence intervals were calculated. ZPH test was used together with a time varying coefficient plots [fitted penalized B-spline curve with 95% CI)] to check for non-proportional hazards. In case of non-proportionality, the variables were taken up in the model as time-varying variable. Separate Kaplan-Meier for assessing OS with a two-sided log rank test were calculated for selected variables in the Cox model.

The analyses to assess predicting factors for OS and PFS were conducted in four steps, because one model with too much variables may not be able to reveal all interesting variables. Hence, four separate, per domain, Cox regression models were fitted: first, a model with all demographic variables, then a second model with disease specific background factors, and a third where the metabolic factors were investigated. Finally, all the remaining best predicting variables were put together in one model (supplementary material). All analyses were conducted with SAS version 9.4 (SAS Institute Ltd., Budapest, Hungary) and Statistica v13.2 (StatSoft Inc., Budapest, Hungary).

## Results

53 patients (42 men and 11 women) were enrolled in the study who received first line therapy for their mCRC. The median time between 1st PET/CT and the 1st chemotherapy was 2.5 weeks (range 1–3 weeks), whereas the 2nd PET/CT occurred always on the 21th day after the first chemotherapy.

28 patients had RAS mutation, while 25 patients had wild-type RAS. Patients with RAS mutation received bevacizumab plus chemotherapy. Nineteen of the wild type RAS patients received cetuximab plus chemotherapy in first line setting; the remaining 6 were treated with bevacizumab plus chemotherapy. The bevacizumab regimen in these cases was chosen to avoid skin toxicity of EGFR inhibitor therapy. Although the literature indicates 40–45% frequency of RAS mutation in CRC [13], in our study 52% of patients had mutant RAS. Several studies have shown that the presence of KRAS mutation increased the risk of relapse and death. A Kaplan-Meier analysis of the two groups indicated a slight but not statistically significant difference in median survival (21.7 for the mutated versus 24 months for wild-type).

The primary tumor was located in the colon in 37 patients and in the rectum in 16 patients (see Table 3). First PET/CT (scan-1) was performed a mean of 9.7 days (range 1–21) before the chemotherapy, scan-2 as early evaluation was carried out on the 21th day after the 1st cycle (so 8 days after the second cycle). The mean number of the detected liver metastases was 8.96. Twenty-eight patients had only liver metastases, whereas the rest (*n* = 25) had extrahepatic metastases as well. In the course of the study 10 patients underwent liver resection, 43 patients died and 10 were alive at the end of follow-up (24 March 2019). The length of follow up time was an average 24 months.

**Table 3:** Demographic and clinical characteristics of the Patients (*n* = 53)

Characteristics	Value
Gender, *n* (%)	
Men	42 (79%)
Women	11 (21%)
Age (years), median	64,7
Primary tumor, *n* (%)	
Colon	37 (70%)
Rectum	16 (30%)
Synchronicity, *n* (%)	
Synchronous	47 (89%)
Metachronous	6 (11%)
Mean number of liver metastases	8,96
Only LM	28
Extrahepatic metastases	25
Primary tumor was not resected	18
Liver resection	10
Type of chemotherapy	
bevacizumab + chemotherapy	35
cetuximab + chemotherapy	18
Molecular type of the tumor	
Wild type	25
Mutant	28

### Metabolic Variables

Table 4 shows the main metabolic parameters on scan-1 and scan-2. Both the SUV_max_, TLG and the SAM parameters (summarized SAM and NSAM) showed a significant decrease from scan-1 to scan-2 in every patient (*p* < 0.0001, for every tested parameter). As expected the SAM values, as they represent a summation of all lesions in the liver show a much larger variation over patients than the single measurements from the highest metabolic lesions (SUV_max_).

**Table 4:** Metabolic parameters before the treatment (scan 1) and after 2 cycles (scan 2)

Parameters	Mean	SD	Min	Max	Statistics*
SUV_max_ 1	11.56	4.75	4.58	24.85	*p* < 0.0001
SUV_max_ 2	7.16	3.89	2.71	27.51	
SAM1	1660.55	2483.69	9.22	10,127.24	*p* < 0.0001
SAM2	436.91	738.56	0	3692.25	
NSAM1	826.95	1376.0	3.35	6430.40	*p* < 0.0001
NSAM2	215.74	402.88	0	2117.12	
TLG1	3040.64	3722.71	31,46	16,800	*p* < 0.0001
TLG2	1048.57	1414.94	0	6071.88	

1-shows the parameters on scan 1; 2-shows the parameters on scan 2

^*^Wilcoxon signed-rank test

### Survival Analysis

We did not find significant correlation between the OS and the gender of the patients (*p* = 0.2327), location of the tumor (left vs. right colon-side *p* = 0.51819), RAS mutation status (*p* = 0.4948), therapeutic choice (VEGFi or EGFRi, *p* = 0.8978), if the metastases were synchron or metachron (*p* = 0.6345). Two disease specific variables were related to OS. Patients without extrahepatic metastases had a statistically significantly longer overall survival compared to patients with extrahepatic metastases (*p* = 0.0172). In addition, the patients who had their liver metastases resected anytime during the observation period, had better overall survival than the patients who were not (*p* = 0.0001).

We did not find significant correlation between the PFS and the gender of the patients (*p* = 0.3599), location of the tumor (left vs. right colon-side *p* = 0.3479), RAS mutation status (*p* = 0.5209), therapeutic choice (VEGFi or EGFRi, *p* = 0.4090), if the metastases were synchron or metachron (*p* = 0.8662). PFS did not improved in patients with no extrahepatic metastases (liver-only disease) vs. patients with extrahepatic disease as well (*p* = 0.714), or in those patients who underwent liver resection due to metastatic disease (*p* = 0.1676).

We performed the detailed Cox-regression analysis for these and the above listed co-variables and the metabolic parameters of the tumors to further assess their relationship with the clinical outcome.

In the Cox model, the liver resection was modelled in a time varying manner, since it cannot be assumed that the HR remains constant before and after the resection. Patients who had their liver metastases resected anytime during the observation period, had better overall survival than the patients who were not (HR 0.949 *p* = 0.0485), but their PFS was not improved significantly (HR 0.964, *p* = 0.0573).

The classification of SAM in terms or metabolic response categories (partial remission, stable disease, progression) was not selected by the model, nor for OS or PFS. The variables SAM2 and NSAM2 were highly correlated and selected by the Cox model as related to OS or PFS, respectively. TLG metabolic variables were not selected as the best predictive factors for OS or PFS.

After the separate analysis per domain, the remaining statistically significant parameters were included into the final model and subjected to a final stepwise selection procedure. Two parameters remained in the final overall survival model. NSAM2 and resection of the liver were statistically significantly predictive of OS (*p* = 0.0056, *p* = 0.0113). For the PFS the SAM2 (*p* = 0.0062) and the absence of extra hepatic metastasis (*p* = 0.0130) were the two best and statistically significant predicting parameters.

This model explained 13.6% of the total variance for OS.

### Additional Analyses for OS - SAM Classification

**Fig. 1:**
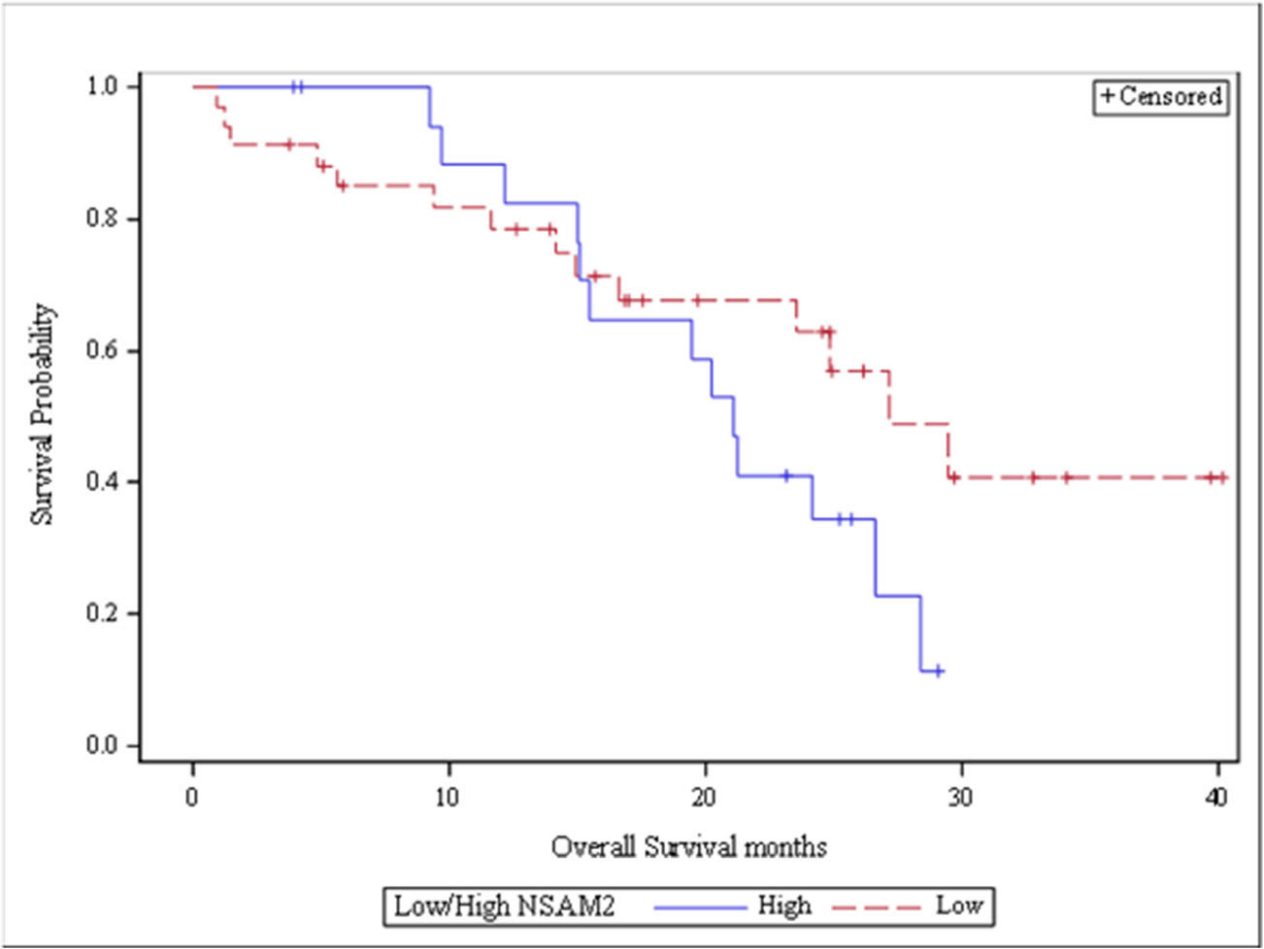
Kaplan-Meier curve of OS by low (<= 150) and high (> 150) NSAM2. (*p* = .0677) difference in OS. The median overall survival was 5.4 months longer in the low NSAM2 group compared to the high group

In order to investigate the effect of NSAM2 a Kaplan-Meier posthoc test was done for OS by categorizing the NSAM2 values. A Kaplan-Meier survival analysis was also done on a low (<= 150) versus high (> 150) strata of NSAM2 (see Fig. 1). Although SAM2 was a predictive variable according to the Cox modeling, it was not statistically significant (*p* = 0.0677) for the low and high NSAM2 strata. The OS between the groups showed a 5.4 month difference. PFS did not show any statistical significant difference between the same strata.

### ROC Analyses for PFS/OS

**Fig. 2:**
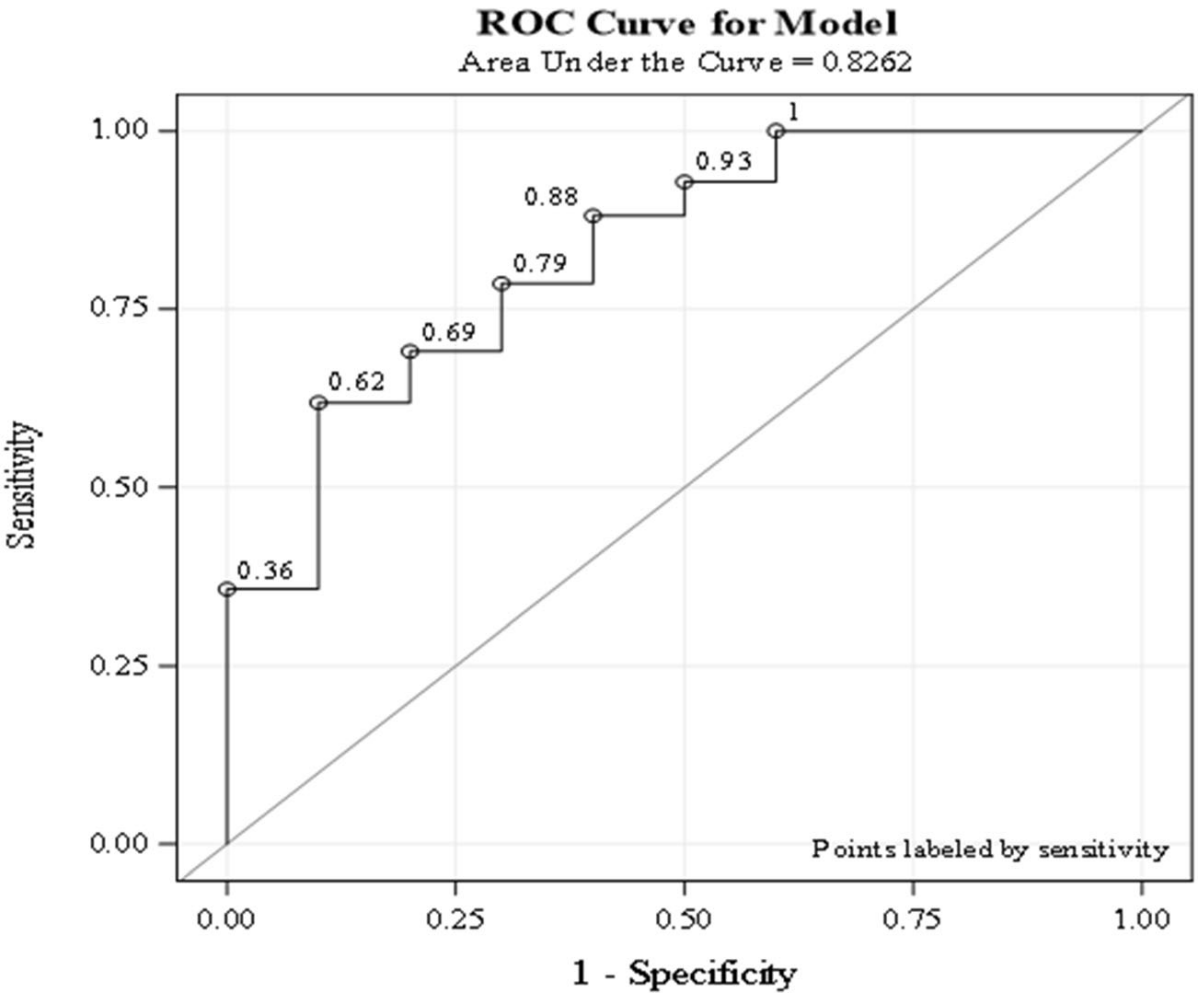
ROC curve for OS using NSAM2 and resection of the liver. The area under the curve (AUC) of these variables is 0.83, whereas each single one generates an AUC of 0.69 and 0.68 for respectively the NSAM2 and resection of the liver

Based on the outcome of the Cox regression a ROC analysis was done for NSAM2 and for resection of the liver for each variables separately and for the combination of the two for OS. In case of OS, the Area Under the Curve (AUC) of NSAM2 and resection of the liver was 0.69 and 0.68, respectively**.** When only those patients were analyzed who went through a liver resection, the ROC AUC value was 0.83. This ROC curve is depicted below (see Fig. 2).

**Fig. 3:**
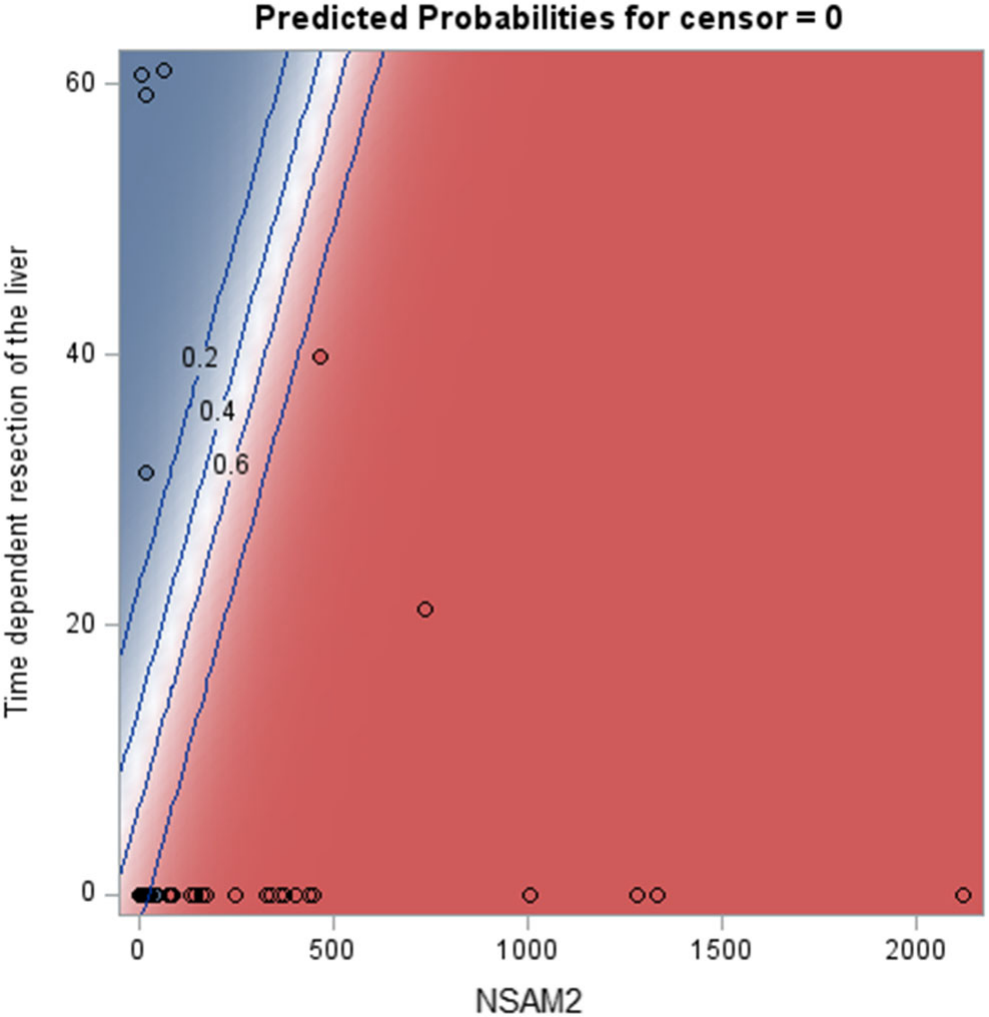
Predicted probability of death based on NSAM2 and the resection of the liver. The black lines indicate the value of the probability

Figure 3 depicts the calculated predicted probability of dying based on the NSAM2 and on the resection of the liver. The blue shaded area indicate a lower than 50% chance of death, whereas the red shaded area indicate the patients with a higher than 50% probability of death. Patients who combine low NSAM2 with a resection of the liver have the best prognosis.

Similar results were obtained for PFS. ROC AUC values of 0.60 were obtained for only liver disease, and ROC AUC of 0.73 for NSAM2. The combination of both yields a ROC AUC of 0.80.

## Discussion

The mechanism of action and toxicities of targeted therapies differ from those of traditional cytotoxic chemotherapy [14]. It also seems like that the measurement of the therapeutic response could also be challenging in case of targeted therapies, therefore the role of metabolic imaging with hybrid modalities is emerging [15–19]. The current study investigated early metabolic treatment response of mCRC patients using PET/CT and tried to assess its value using different metabolic parameters for prediction of OS and PFS. There are several previous investigations assessed early metabolic response after first or second cycles of therapy [15–19]. Most of these studies, however, used heterogeneous patient population, as indicated by more than three different types of chemotherapy used, and by the vast majority of non-chemo-naive patients included. Moreover, these studies included a low number of patients, and some of them were retrospective [15–19].

Hendlisz et al. [20] reported results of 40 mCRC patients receiving different types of chemotherapy as first or second line. They found that early metabolic response was associated with OS. In comparison, our study had a more homogeneous patient group, none of whom received previously chemotherapy for metastatic disease, and all of them received targeted therapy.

In our prospective trial several metabolic parameters and other factors were examined (see Table 1).

We do not find significant correlation between survival and routinely examined patients’ characteristics such as age or BMI. Moreover we do not find significant correlation with the RAS mutation status and survival in this patient group. A separate Kaplan-Meier comparison of wild type patients with mutated patients did not produce a statistically significant different survival estimate, but the median survival differences between the two strata amounted to 2.3 months. The reason for these results could based on the current debate about the best treatment choice for mCRC based on RAS mutational analysis and tumor location [21–23].

From the tested clinical and metabolic parameters (based on PET/CT examinations) only NSAM2 and resection of the liver have a statistically significant predictive effect on OS.

PFS did not improve in patients with liver-only disease vs. patients with extrahepatic metastases (*p* = 0.714).

Additionally, PFS was not improved in those patients who underwent liver resection due to metastatic disease (*p* = 0.1676). Nonetheless, PFS in our cases meant the progression during the first-line chemotherapy. Thereafter we used another 2–4 lines of chemotherapy, several were combined by targeted therapy as well. Therefore, the overall clinical outcome and the overall survival of those patients who underwent liver resection could became favorable compared to those who did not. This was true in our patient group, as well – we found significantly longer OS in patients with a successful resection of liver metastases (*p* = 0.0001). This differences remained significant even if we performed a Cox regression to rule out the bias which can be cause bay the timing of the resections. Additionally, those patients who had extrahepatic disease, OS was also significantly worse compared to liver only disease (*p* = 0.0172).

During the PET/CT response evaluation we compared several metabolic parameters to measure the clinical response. Both SUVmax, TLG, SAM and NSAM showed significantly the decrease in the metabolic activity of the tumors to the first line therapy. However, only NSAM2 had a highly statistically significant effect on the clinical outcome. The Kaplan-Meier investigated strata defined as higher NSAM2 than 150, or lower and equal than 150 showed a 5.4 month median OS difference. This, however, was not significant. For both OS and PFS the metabolic parameter of NSAM2 or SAM2 appears to be strong predictors of patient outcome according to the Cox analysis, but the Kaplan-Meier analysis was not show significant survival differences. The reason for this is most likely that the Cox regression has more power in handling continuous co-variates than the discrete Kaplan-Meier two strata approach. The limited group size when creating arbitrary strata and the impact of the relatively high number of censored observations per strata may have accentuated the differences between both techniques. Based on this we can state, that the relatively low number of patients in some of the subgroups could be the main limitation of our study.

## Conclusion

Our prospective study evaluated the role of FDG-PET/CT based metabolic parameters measured after the second therapeutic cycle during the first line treatment of mCRC in the prediction of therapeutic effectiveness and survival. Although we found similar results as described by Mertens et al. [11], but in our study SUV_max_, TLG, and the percentage reduction of baseline metabolic parameters did not showed significant correlation with therapeutic effect or with survival. Nonetheless, the more complex values of SAM and NSAM were good predictors of OS and PFS, if measured after the second therapeutic cycle.

In conclusion, our results showed that in early response evaluation with FDG-PET/CT acquired metabolic variables only SAM2 and NSAM2 could have a role in predicting OS and PFS and guiding further treatment.

## Electronic supplementary material

ESM 1 (PNG 8 kb)

ESM 2 (PNG 8 kb)

ESM 3 (PNG 11 kb)

## Abbreviations

AUC: Area Under the Curve
BG: Background
FDG-PET/CT: ^18^F-Fluorodeoxyglucose Positron Emission Tomography and Computed Tomography
HR: Hazard Ratio
mCRC: metastatic Colorectal Cancer
OS: Overall Survival
PFS: Progression-Free Survival
ROC: Receiver Operating Characteristic analysis
SUV_max_: maximum of the Standardized Uptake Volume
SAM: Standardized Added Metabolic Activity
TLG: Total Lesion Glycolysis
VOI: Volume Of Interest
