# Supplementary figures and images for: Metabolic Parameters as Predictors for Progression Free and Overall Survival of Patients with Metastatic Colorectal Cancer
Source: Pathol Oncol Res. 2020 Jul 13;26(4):2683–91. doi: 10.1007/s12253-020-00865-5 (PMC7772167; doi:10.1007/s12253-020-00865-5)

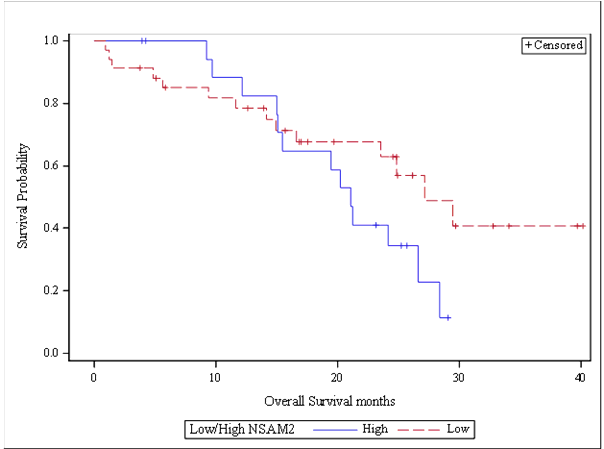

Supplement: Supplementary file 1 — (PNG 8 kb) [file 12253_2020_865_MOESM1_ESM.png]

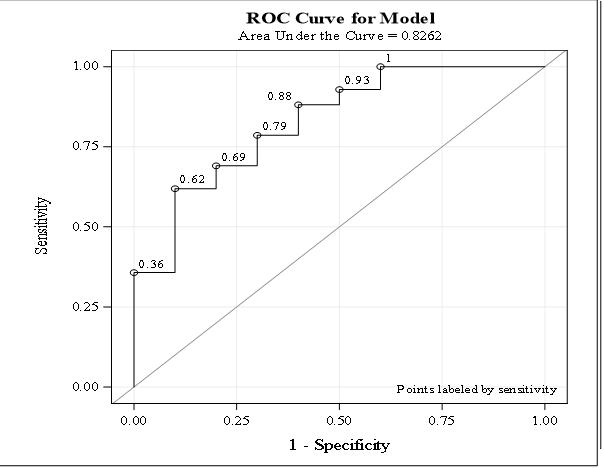

Supplement: Supplementary file 2 — (PNG 8 kb) [file 12253_2020_865_MOESM2_ESM.png]

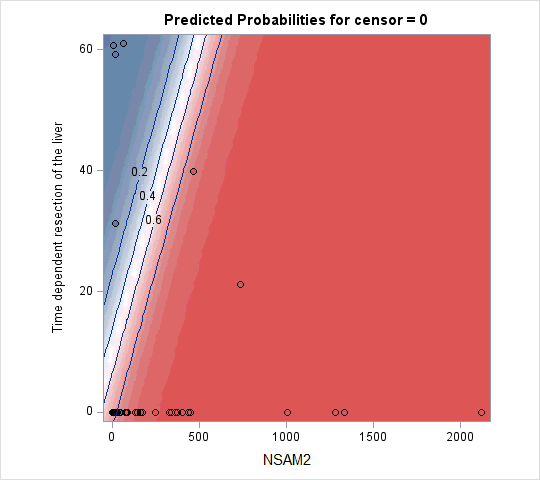

Supplement: Supplementary file 3 — (PNG 11 kb) [file 12253_2020_865_MOESM3_ESM.png]
